# Is exclusive breastfeeding for six-months protective against pediatric tuberculosis?

**DOI:** 10.1080/16549716.2020.1861922

**Published:** 2021-01-03

**Authors:** Juan A. Flores, Julia Coit, Milagros Mendoza, Segundo R. Leon, Kelika Konda, Leonid Lecca, Molly F. Franke

**Affiliations:** a Escuela Profesional de Tecnología Médica, Universidad Privada San Juan Bautista, Lima, Peru; b Facultad de Salud Pública, Universidad Peruana Cayetano Heredia, Lima, Peru; c Department of Global Health and Social Medicine, Harvard Medical School, Boston, MA, USA; d Direction of Research, Socios En Salud at Partners in Health, Lima, Peru; e Division of Infectious Diseases, University of California, Los Angeles, CA, USA; f Center for Interdisciplinary Studies in Sexuality, AIDS and Society, and Laboratory of Sexual Health, Universidad Peruana Cayetano Heredia, Lima, Peru

**Keywords:** *Mycobacterium tuberculosis*, child, risk factor, breastfeeding, infant

## Abstract

Experts recommend exclusive breastfeeding from birth to six months because it protects against deadly childhood illness, including respiratory tract infections and diarrhea. We hypothesized that exclusive breastfeeding would decrease the risk of active tuberculosis (TB) in children. We analyzed cross-sectional data from 279 children in Lima, Peru aged 6 to 59 months with TB symptoms and a close adult contact with TB. Mothers self-reported breastfeeding, and children were evaluated for TB per national guidelines. To quantify the association between exclusive breastfeeding and TB, we estimated prevalence ratios using a generalized linear model with a log link, binomial distribution, and robust variance. Twenty-two percent of children were diagnosed with TB and 72% were exclusively breastfed for six months. We found no evidence that six months of exclusive breastfeeding was associated with TB disease in either bivariate analyses (prevalence ratio [PR] = 1.5; 95%CI = 0.8–2.5) or multivariable analyses adjusting for sex and socioeconomic status (adjusted PR = 1.6; 95%[CI] = 0.9–2.7). In post hoc analyses among children whose close TB contact was their mother, we found evidence of a weak positive association between breastfeeding and TB (aPR = 2.1; 95%[CI] = 0.9–4.9). This association was not apparent among children whose close contact was not the mother (aPR = 1.2; 95%[CI] = 0.6–2.4). Our results raise the possibility that children who are breastfed by mothers with TB may be at increased risk for TB, given the close contact. Due to the cross-sectional study design, these results should be interpreted with caution. If these findings are confirmed in longitudinal analyses, future interventions could aim to minimize TB transmission from mothers with TB to breastfeeding infants.

## Background

Tuberculosis (TB) is an important cause of morbidity and mortality among children. In 2018, 1.12 million children under 15 years old became ill with TB; nearly 25% of whom died of the disease [1]. Neonatal TB mortality can be as high as 40–60% [2–5] and mothers with active TB can transmit the infection to their infants in-utero or post-partum [6].

Globally, 41% of infants are exclusively breastfed for the first six month of life, as recommended by the World Health Organization (WHO) [7]. However, this rate is far lower than the 2030 global target of 70% [8]. Exclusive breastfeeding means that an infant receives only breastmilk and no other food or liquids, including water; the only exceptions are oral-rehydration salts or medications [9]. Evidence supporting this recommendation suggests that breast milk contains bioactive components and anti-inflammatory properties that promote immune development [10,11].

Exclusive breastfeeding protects against common pediatric illnesses including diarrhea, gastro-enteritis, otitis media and respiratory tract infections including pneumonia [12–15]. However, it is unknown whether it affords protection against TB. Given its role in immune system maturation, response and memory [16], we hypothesized that exclusive breastfeeding during the first 6 months of life may confer protection against TB. To explore this hypothesis, we quantified the association between exclusive breastfeeding and pediatric TB in children aged 6 to 59 months of age.

## Methods

### Study design and setting

We conducted a secondary analysis of cross-sectional data from a prospective pediatric TB diagnostics study (the ‘parent study’) in Lima, Peru that enrolled children under 15 years old [17–20] who were undergoing evaluation for TB in accordance with Peruvian guidelines [21]. In 2016, Peru just met the 2030 WHO global breastfeeding target with 70% exclusive breastfeeding coverage [8,22].

### Study population and data collection

**Figure 1.**
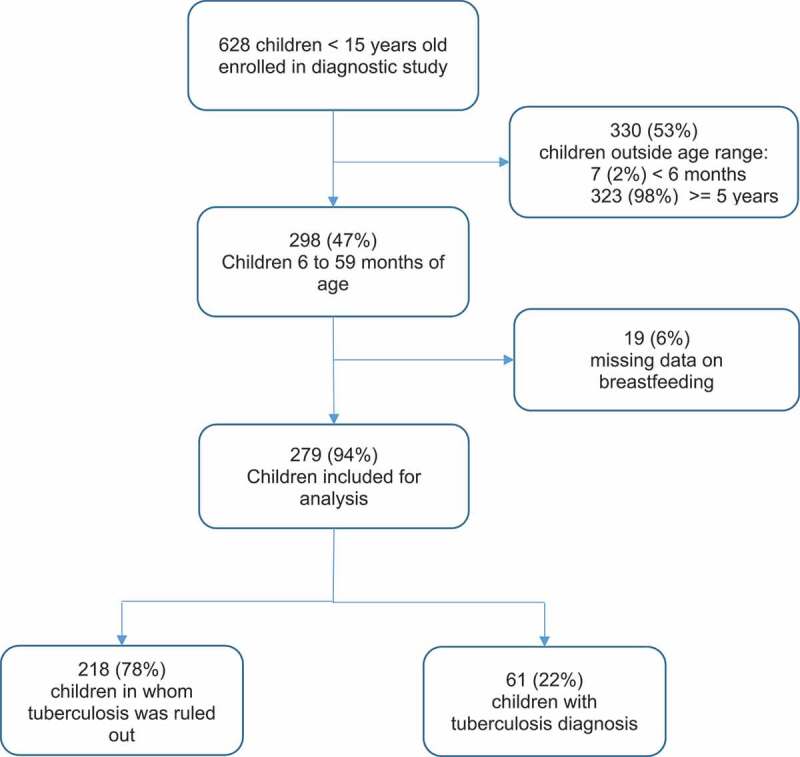
Flowchart of enrollment and exclusions

Between May 2015 and February 2018, children were recruited into the parent study from 49 health centers in Lima. To maximize TB case yield, eligible children were those with a history of contact with an adult with pulmonary TB within the previous two years and with one or more symptoms compatible with TB, as defined by an expert panel [23]: persistent, unremitting, and unexplained cough for >2 weeks, unexplained weight loss, unexplained fever for >1 week, or unexplained fatigue or lethargy. Study staff implemented standardized questionnaires to collect clinical, social and demographic data via a face-to-face interview with caregivers. Figure 1 shows the flowchart for inclusion in this analysis.

### Data analysis

Children classified as having TB were those who had a positive culture result or, in its absence, a clinical diagnosis made on the basis of chest x-ray, symptomatology and tuberculin skin testing, by a pediatric pulmonologist. Exclusive breastfeeding was determined based on maternal report and analyzed as dichotomous (6 months vs. less than six month) to reflect current WHO recommendations and continuous (in months) to examine a dose response.

We estimated prevalence ratios using a generalized linear model with a log link, binomial distribution and robust variance to quantify the association between exclusive breastfeeding and TB. Given the small dataset, we aimed for a parsimonious multivariable model. We adjusted for socioeconomic index (SEI) and sex, which were identified a priori as potential confounders. Then, from a list of other biologically plausible potential confounders (e. age in continuous [24], whether the contact was the child’s mother, shared a bed with the child, smoked cigarettes or drank alcohol [25,26], we identified those associated with TB at a p-value threshold of ≤0.20 and adjusted for them in the final model. The SEI variable was constructed using a principal component analysis (PCA) based on the household characteristics and family possessions. Because we expected the SEI to predict TB risk in a linear fashion, we modelled it as a continuous linear variable (supplementary material 1). When the binomial model failed to converge, we used a Poisson distribution.

We conducted post hoc stratified analyses to examine potential effect modification by age and whether the child’s close contact with TB was his/her mother. With regard to age, we hypothesized that any protective effect of breastfeeding on TB might be most apparent in children less than 24 months of age; the immune system is weakest at this time [27] and exposure to breastfeeding is likely to be more proximal to TB exposure in younger children than in older children. Whether the mother was the child’s close contact with TB is relevant because a protective effect of breastfeeding could be attenuated by increased exposure to *Mycobacterium tuberculosis* during breastfeeding. Data analyses were performed using Stata v16.0 (StataCorp, College Station, TX).

## Results

Of the 628 children enrolled in the parent study, 298 (47%) were between 6 and 59 months of age and therefore eligible for inclusion in the present analysis. Of these, 19 (6%) lacked data on breastfeeding and were excluded (Figure 1). Of the 279 children included, 61 (22%) were diagnosed with TB, of whom 12 (20%) had a positive culture. Among all children included, 72% (200/279) were exclusively breasted for six months. Among those who were exclusively breastfed for less than six months or not at all (n = 79), the median duration of exclusive breastfeeding was 4 months (IQR = 2–5). Patient characteristics are described in Table 1.

**Table 1.** Characteristics of children exposed to exclusive breastfeeding (N = 279)*

			Exclusive breastfeeding for six months	
		Total	Yes (N = 200)	No (N = 79)
Variables		n (%)	n (%)	n (%)
**Age (months)**				
	< 24	112 (40)	80 (40)	32 (41)
	24 to 59	167 (60)	120 (60)	47 (59)
**Sex**				
	Male	149 (53)	103 (52)	46 (58)
	Female	130 (47)	97 (48)	33 (42)
**Index case is the mother (N = 275)**				
	Yes	96 (35)	63 (32)	33 (42)
	No	179 (65)	133 (68)	46 (58)
**Index case shares bed with children (N = 275)**				
	Yes	118 (43)	80 (41)	38 (48)
	No	157 (57)	116 (59)	41 (52)
**Index case drinks (N = 274)**				
	Yes	171 (62)	126 (64)	45 (58)
	No	103 (38)	70 (36)	33 (42)
**Index case smokes (N = 273)**				
	Yes	110 (40)	84 (43)	26 (33)
	No	163 (60)	111 (57)	52 (67)
**SEI (N = 278)**				
	Median (IQR)	−0.05 (2.8)	−0.05 (2.3)	−0.13 (2.4)
**TB diagnosed**				
	Yes	61 (22)	48 (24)	13 (16)
	No	218 (78)	152 (76)	66 (84)

*Unless otherwise noted.

Abbreviation: IQR = interquartile range, TB = tuberculosis, SEI = Socioeconomic Index.

### Bivariate and multivariate analyses

We found no association between exclusive breastfeeding and active TB in the bivariate analysis (prevalence ratio [PR] = 1.5; 95%[CI] = 0.8–2.5). None of the potential confounders identified *a priori* were associated with TB at the defined threshold of ≤0.20; therefore, multivariable analyses were adjusted for sex and SEI. In multivariate analyses, children who were exclusively breastfed for six months had a 60% higher prevalence of active TB compared to children who were not exclusively breastfed; however, the confidence interval was wide (adjusted PR = 1.6; 95%[CI] = 0.9–2.7; p = 0.111). There was no association when we evaluated exclusive breastfeeding as a continuous variable in months (adjusted PR = 1.0; 95%[CI] = 0.9–1.1, p = 0.983).

### Effect modification by age and maternal TB history

Stratifying by age, we found that children between 6 and 23 months of age who were exclusively breastfed for six months had twice the prevalence of TB as compared to those that were not exclusively breastfed for 6 months. This association was greatly attenuated among older children; however confidence intervals were wide and significance testing suggested that chance was a likely explanation for these differences (aPR = 2.2; 95%[CI] = 0.8–5.7 in children aged 6–23 months old versus aPR = 1.2; 95%[CI] = 0.6–2.5 in children over 23 months; p-value for interaction = 0.390). Similarly, among children whose close contact was their mother, we found that six months of exclusive breastfeeding was associated with twice the prevalence of TB, and this was not observed among children whose contact was someone other than their mother (aPR = 2.1;95%[CI] = 0.9–4.9 versus aPR = 1.2;95%[CI] = 0.6–2.4, respectively; p-value for interaction = 0.380) (Table 2). Once again, confidence intervals were wide, and p-values for interaction suggested that chance was one likely explanation for these differences.

**Table 2.** Associations between exclusive breastfeeding for six months and TB in children aged 6 to 59 months

Models‡	N	PR	CI 95%	p value	aPR	CI 95%	p value
**All children**	279	1.5	0.8–2.5	0.183	1.6**	0.9–2.7	0.111
**Stratified by age**							
6 to 23 months of age	112	2.2	0.8–5.9	0.118	2.2**	0.8–5.7	0.109
24 to 59 months of age	167	1.1	0.6–2.2	0.722	1.2	0.6–2.5	0.540
**Stratified by contact**							
Contact was mother	96	2.0	0.8–4.9	0.132	2.1	0.9–4.9	0.097
Contact was not mother	179	1.2	0.6–2.4	0.672	1.2**	0.6–2.4	0.640

‡Analyses were conducted using generalized linear binomial regression models, which adjusted for sex and socioeconomic index.

**These variables had missing data for one child.

Abbreviation: PR = Prevalence ratio; aPR = Adjusted prevalence ratio; CI = Confidence interval.

## Discussion

We found no evidence of a protective association between exclusive breastfeeding and TB in children between 6 and 59 months of age. Among children aged 6 to 23 months and children whose TB contact was their mother, we found weak evidence that 6 months of exclusive breastfeeding was positively associated with TB. This raises the possibility that exposure to TB during breastfeeding plays a more determinant role in the child’s risk of disease than the theoretical protection against TB that six months of exclusive breastfeeding may grant.

Breastfeeding is a cheap and healthy way to feed a baby. The WHO recommends that breastfeeding continue irrespective of the TB status of the mother when she is on appropriate anti-TB treatment for active disease [28]. Breastfeeding cessation is recommended if a woman has active tubercular breast lesions or tubercular mastitis [6]. In cases where a mother has active TB and is breastfeeding her infant, general protective measures, such as masks, could be used, particularly in the early phases of treatment [6,28]. Counseling breastfeeding women with TB on the recommendations and risks and ensuring timely and appropriate TB-treatment will contribute to TB prevention in infants [29]. Future longitudinal research on TB in pregnant and breastfeeding women will be important to more fully understand the relationship between exclusive breastfeeding and TB and potential interventions that will maximize health and well-being for them and their infants.

Among the limitations of this study are the cross-sectional design and lack of information on the timing of breastfeeding relative to exposure to a close contact with TB and limited data on other potential confounders.

Although we found no significant association between TB and exclusive breastfeeding in children aged 6 to 59 months, limitations in our study design preclude us from discarding the possibility that exclusive breastfeeding may afford protection against TB. The benefits of breastfeeding in children are indisputable [10–15]. In young children whose close contact was their mother and in children between 6 and 23 months of age, we found weak evidence that exclusive breastfeeding was associated with an elevated risk of TB, underscoring the critical importance of rapid diagnosis and treatment of pregnant women and breastfeeding mothers with TB.

## Supplementary Material

Supplemental Material

## Paper context

Despite not having found any association between breastfeeding and TB in children aged 6 to 59 months overall, we found weak evidence that exclusive breastfeeding was associated with an elevated risk of TB among children whose close contact was their mother. These associations should be evaluated in longitudinal studies. Timely diagnosis and treatment of women with TB, along with counseling for those who plan to breastfeed, will contribute to TB prevention in infants.
