# Supplementary material for: Is exclusive breastfeeding for six-months protective against pediatric tuberculosis?
Source: Glob Health Action. 2021 Jan 3;14(1):1861922. doi: 10.1080/16549716.2020.1861922 (PMC7782219; doi:10.1080/16549716.2020.1861922)
Supplement: Supplemental Material [file ZGHA_A_1861922_SM9332.docx]

Supplementary material 1. Categorical variables of housing and family possessions included in PCA analyses to calculate socioeconomic index (n=278)*

| **Does your household have any of the following?** | **Total** | **TB diagnosed** | |
| --- | --- | --- | --- |
|  | **n (%)** | **Yes (N=61)**  **n (%)** | **No (N=217)**  **n (%)** |
| **Indoor bathroom** | 242 (87) | 46 (75) | 196 (90) |
| **Cellphone** | 241 (87) | 50 (82) | 191 (88) |
| **Computer** | 66 (24) | 14 (23) | 52 (24) |
| **Microwave** | 91 (33) | 19 (31) | 72 (33) |
| **Conventional Phone** | 65 (24) | 10 (16) | 55 (25) |
| **Drinkable water** | 250 (90) | 49 (80) | 201 (93) |
| **Refrigerator** | 198 (71) | 36 (59) | 162 (75) |
| **Television-artefact** | 273 (98) | 59 (97) | 214 (99) |
| **Motor vehicle** | 31 (11) | 5 (8) | 26 (12) |
| **Washing machine** | 132 (48) | 30 (49) | 102 (47) |
| **>1 windows to exterior** | 147 (53) | 29 (48) | 118 (54) |
| **>1 rooms** | 211 (76) | 45 (74) | 166 (77) |
| **Socioeconomic Index** | -0.05 (2.8)^£^ | -0.15 (3.7)^£^ | -0.02 (2.4)^£^ |
|  |  |  |  |

* One participant was missing data for all variables.

^£^ Median (IQR)

Abbreviation: PCA= principal component analysis
